# Activation of Pancreatic Acinar FXR Protects against Pancreatitis via Osgin1-Mediated Restoration of Efficient Autophagy

**DOI:** 10.34133/2022/9784081

**Published:** 2022-11-02

**Authors:** Yufan Zheng, Wenrui Sun, Zhengyang Wang, Jiaying Liu, Cong Shan, Chenxi He, Borui Li, Xiao Hu, Wenjia Zhu, Liyan Liu, Fei Lan, Changtao Jiang, Chao Zhao, Xiaobo Li, Ning Sun

**Affiliations:** ^1^Wuxi School of Medicine, Jiangnan University, Jiangsu, China; ^2^Department of Physiology and Pathophysiology, State Key Laboratory of Medical Neurobiology, School of Basic Medical Sciences, Fudan University, Shanghai, China; ^3^Department of Pathology, The First Affiliated Hospital of Zhengzhou University, Zhengzhou, China; ^4^Shanghai Key Laboratory of Medical Epigenetics, International Laboratory of Medical Epigenetics and Metabolism, Ministry of Science and Technology, Institutes of Biomedical Sciences, Fudan University and Key Laboratory of Carcinogenesis and Cancer Invasion, Ministry of Education, Liver Cancer Institute, Zhongshan Hospital, Fudan University, Shanghai, China; ^5^General Practice/International Medical Care Center, Shanghai General Hospital, Shanghai Jiao Tong University School of Medicine, Shanghai, China; ^6^Department of Physiology and Pathophysiology, School of Basic Medical Sciences, Peking University, Key Laboratory of Molecular Cardiovascular Science, Ministry of Education, Beijing, China

## Abstract

Pancreatitis is the leading cause of hospitalization in gastroenterology, and no medications are available for treating this disease in current clinical practice. FXR plays an anti-inflammatory role in diverse inflammatory diseases, while its function in pancreatitis remains unknown. In this study, we initially observed a marked increase of nuclear FXR in pancreatic tissues of human patients with pancreatitis. Deleting the FXR in pancreatic acinar cells (FXR^acinar*Δ*/*Δ*^) led to more severe pancreatitis in mouse models of caerulein-induced acute and chronic pancreatitis, while the FXR agonist GW4064 significantly attenuated pancreatitis in caerulein or arginine-induced acute pancreatitis and caerulein-induced chronic pancreatitis. FXR deletion impaired the viability and stress responses of pancreatic exocrine organoids (PEOs) in vitro. Utilizing RNA-seq and ChIP-seq of PEOs, we identified *Osgin*1 as a direct target of FXR in the exocrine pancreas, which was also increasingly expressed in human pancreatitis tissues compared to normal pancreatic tissues. Pancreatic knockdown of *Osgin*1 by AAV-pan abolished the therapeutic effects of FXR activation on pancreatitis, whereas pancreatic overexpression of *Osgin*1 effectively alleviated caerulein-induced pancreatitis. Mechanistically, we found that the FXR-OSGIN1 axis stimulated autophagic flux in the pancreatic tissues and cell lines, which was considered as the intrinsic mechanisms through which FXR-OSGIN1 protecting against pancreatitis. Our results highlight the protective role of the FXR-OSGIN1 axis in pancreatitis and provided a new target for the treatment of this disease.

## 1. Introduction

Pancreatitis is an inflammatory disorder of the pancreas with considerable morbidity, mortality, and socioeconomic burden [1]. Acute pancreatitis (AP), with a global incidence of approximately 0.034% [2], usually starts from acinar cell death and quickly progresses to systemic inflammation that requires emergency management. AP patients usually complain of severe acute pain in left upper abdomen and are at a high risk of developing systemic inflammation or multiple organ dysfunction (MODS). About 50 out of every 100,000 persons in the world suffer from chronic pancreatitis (CP) [3], manifesting slowly developed pancreatic inflammation. Some patients with AP will develop into CP due to recurrent pancreatitis [4]. In the pathogenesis of CP, repetitive episodes of pancreatic inflammation cause normal tissues being gradually replaced by fibrotic tissues, leading to chronic pain, pancreatic exocrine and endocrine dysfunction, and reduced life expectancy [5]. To date, there is no specific pharmacological therapeutics for both acute and chronic pancreatitis in the clinic.

The major cause of AP, accounting for up to 40% of the cases, is gallstone disease [6]. When the common bile duct is obstructed, biliary-pancreatic reflux causes pancreatic acinar cell death and tissue inflammation. Previous studies demonstrated that one of the major bile acid receptors, cell surface G protein-coupled bile acid receptor-1 (GPBAR1), is involved in the pathogenesis of AP [7, 8]. Besides GPBAR1, there is another key bile acid receptor, namely, FXR (also called nuclear receptor subfamily 1 group H member 4, NR1H4), that is present in the pancreas [9–11]. Whether FXR plays a role in the pathogenesis of pancreatitis remains unclear.

FXR is a ligand activated transcription factor. Upon activation, FXR predominately binds inverted repeats spaced by one nucleotide (IR-1) on its target genes [12] and plays critical roles in the regulation of glucose, lipid, bile acid, and amino acid metabolism within liver and intestine [13, 14]. Recent studies also showed that FXR was key for various inflammatory diseases. FXR promoted the transactivation of SHP to inhibit c-Jun-mediated osteopontin production in NKT cells and mitigated acute hepatitis [15]. By antagonizing the activator protein-1 (AP-1), stat3, and nuclear factor-kappa B signaling pathways, FXR alleviates inflammation and protects impaired intestinal epithelial barrier integrity in inflammatory bowel disease (IBD) [16]. In addition to transcriptional regulation, FXR was found to directly interact with NLRP3 and caspase 1, negatively regulating the NLRP3 inflammasome in cholestasis-associated sepsis [17]. These studies give us a clue that FXR may participate and play an anti-inflammatory role in the pathogenesis of pancreatitis.

In the present study, we observed that FXR was dramatically increased in the nuclei of pancreatic acinar cells in human patients with pancreatitis. This observation drove us to generate mice with pancreatic acinar-specific deletion of FXR (FXR^acinar*Δ*/*Δ*^) to further investigate the functional role of FXR in pancreatitis. We found that activation of pancreatic acinar FXR protected against pancreatitis. Activation of FXR by the agonist GW4064 was able to alleviate the disease in different mouse pancreatitis models. We further identified that oxidative stress induced growth inhibitor 1 (*Osgin*1) was a major direct FXR target gene in pancreatic exocrine cells and mediated the protective effects of FXR activation on pancreatitis through restoration of efficient autophagy.

## 2. Results

### 2.1. Nuclear FXR Level Was Elevated in Human and Murine Pancreatitis Tissues

**Figure 1:**
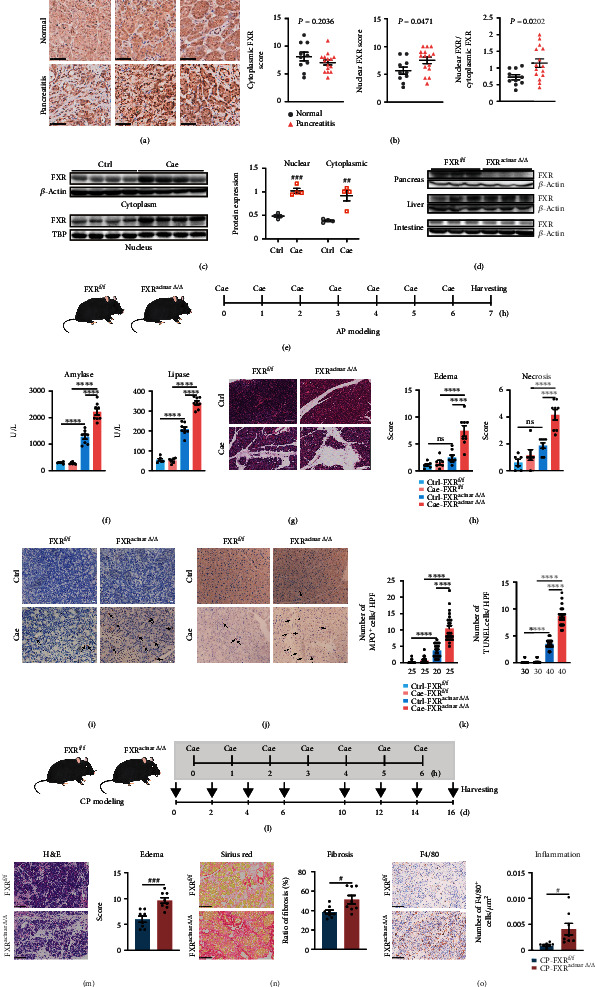
Nuclear FXR level was elevated in pancreatitis and deletion of pancreatic acinar FXR led to more severe pancreatitis. (a) Representative images of FXR immunohistochemical staining from three pancreatitis tissues and three normal pancreatic tissues in human patients. Scale bars, 50 *μ*m. (b) Scoring for the FXR levels in cytoplasm and nucleus of human pancreatic tissues as well as the ratio of nuclear and cytoplasmic FXR scores (normal tissues, *n* = 10; pancreatitis tissues, *n* = 15). (c) Immunoblot and its statistics of nuclear and cytoplasmic FXR in pancreatitis tissues and normal tissues from mice. (d) Immunoblot of FXR in pancreas, liver, and intestine from FXR^f/f^ and FXR^acinar*Δ*/*Δ*^ mice. (e) Schedule for AP modeling in FXR^f/f^ and FXR^acinar*Δ*/*Δ*^ mice. (f) Serum amylase and serum lipase levels of FXR^f/f^ and FXR^acinar*Δ*/*Δ*^ mice with or without caerulein challenge. (g) Representative H&E images of the pancreas of FXR^f/f^ and FXR^acinar*Δ*/*Δ*^ mice with or without caerulein challenge. Scale bars, 100 *μ*m. (h) Evaluation for tissue edema and necrosis in the pancreas (FXR^f/f^ and FXR^acinar*Δ*/*Δ*^ mice treated with saline: *n* = 6; FXR^f/f^ and FXR^acinar*Δ*/*Δ*^ mice treated with caerulein: n = 8). (i, j) Representative images of MPO (i) and TUNEL (j) immunostaining of pancreatic tissues from each group. Scale bars, 20 *μ*m. (k) Statistics plot was performed the number of MPO and TUNEL-positive acinar cells per high-power field (HPF). Five HPFs per individual. The number of HPFs used for the summarized plots of MPO and TUNEL immunostaining are indicated. (l) Schedule for CP modeling in FXR^f/f^ and FXR^acinar*Δ*/*Δ*^ mice. (m) Representative images of H&E staining and tissue edema scoring of pancreatic tissues from repeated caerulein-challenged FXR^f/f^ (*n* = 8) and FXR^acinar*Δ*/*Δ*^ (*n* = 8) mice. Scale bars, 250 *μ*m. (n) Representative images of Sirius Red staining and evaluation of fibrosis of pancreatic tissues. Scale bars, 50 *μ*m. (o) Representative images of F4/80 immunostaining and number of F4/80^+^ cells/*μ*m^2^ of pancreatic tissues. Scale bars, 100 *μ*m. Data are presented as the mean ± SEM. ^#^*P* < 0.05, ^##^*P* < 0.01, and ^###^*P* < 0.001 via unpaired Student's *T* test. ^∗∗∗∗^*P* < 0.0001 via one-way ANOVA. ns: not significant.

To see whether FXR is involved in pancreatitis, we assessed FXR expression in pancreatic tissue sections from patients with pancreatitis by immunohistochemical (IHC) staining, with normal pancreatic tissues from patients with pancreatic cancer as the control. FXR was mainly expressed in the cytoplasm of normal pancreatic tissues. There was no difference in cytoplasmic FXR levels between pancreatitis and normal pancreatic tissues (Figures 1(a) and 1(b)). However, nuclear FXR was markedly increased in pancreatitis tissues compared to that in normal pancreatic tissues (Figures 1(a) and 1(b)). Next, we assessed FXR expression in murine models of caerulein-induced pancreatitis. Dramatically increased nuclear FXR expression was also observed in pancreatitis tissues (Figure 1(c)). These data demonstrated that FXR was upregulated in human and murine pancreatitis tissues.

### 2.2. Pancreatic Acinar Deletion of FXR Aggravated Pancreatitis in Murine AP and CP Models

We next generated FXR^acinar*Δ*/*Δ*^ mice (Figure 1(d) and Figure S1) to further investigate the possible function of FXR on pancreatitis (Figure 1(e)). Pancreatic acinar deletion of FXR did not directly induce alterations in pancreatic histology and exocrine function. However, when challenged with caerulein, FXR^acinar*Δ*/*Δ*^ mice presented more severe pancreatitis phenotypes compared with FXR^f/f^ mice, including elevated serum amylase and lipase level (Figure 1(f)), more severe tissue edema and necrosis (Figures 1(g) and 1(h)), and more neutrophil infiltrations and apoptotic acinar cells in pancreas (Figures 1(i)–1(k)). Moreover, we challenged FXR^acinar*Δ*/*Δ*^ and FXR^f/f^ mice with repeated caerulein injection (Figure 1(l)). The results showed that FXR deletion led to a significant increase in CP-associated tissue edema, fibrosis, and inflammation infiltration (Figures 1(m)–1(o)). These findings suggested a protective role of FXR in pancreatitis.

### 2.3. GW4064, an FXR Agonist, Alleviated Pancreatitis in Murine Models

**Figure 2:**
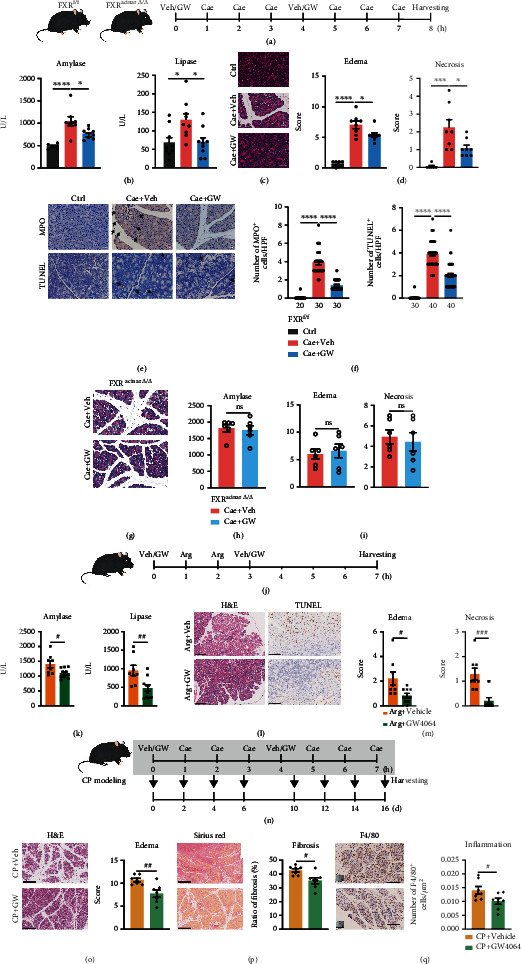
Administration of FXR agonist GW4064 in mice alleviated pancreatitis. (a) Scheme for GW4064 administration in caerulein-induced pancreatitis murine models. (b) Serum amylase levels of mice treated with saline + vehicle (*n* = 6), caerulein + vehicle (*n* = 8), or caerulein + GW4064 (*n* = 9). (c) Representative H&E images of pancreatic tissues. (d) Scoring for pancreatic tissue edema and necrosis (mice treated with saline + vehicle: *n* = 6; mice treated with caerulein + vehicle: *n* = 8; mice treated with caerulein + GW4064: *n* = 8). (e) Representative images of MPO and TUNEL immunostaining of pancreatic tissues. Scale bars, 20 *μ*m. (f) Statistics for the number of MPO and TUNEL-positive acinar cells per HPF. Five HPFs per individual. The number of HPFs used for the summarized plots of MPO and TUNEL immunostaining are indicated. (g) Representative H&E images of pancreatic tissues from FXR^acinar*Δ*/*Δ*^ mice treated with caerulein. Scale bars, 20 *μ*m. (h, i) Serum amylase levels (h) and scoring for tissue edema and necrosis (i) of FXRacinar*Δ*/*Δ* mice treated with caerulein. *n* = 6 in each group. (j) Scheme for GW4064 administration in L-arginine-induced AP murine models. (k) Serum amylase and lipase levels of mice treated with L − arginine + vehicle (*n* = 8) or L − arginine + GW4064 (*n* = 10). (l) Representative images of H&E staining and TUNEL immunostaining of pancreatic tissues. Scale bars, 100 *μ*m. (m) Scoring for pancreatic tissue edema and necrosis (mice treated with L − arginine + vehicle, *n* = 8. Mice treated with L − arginine + GW4064, *n* = 10). (n) Scheme for GW4064 administration in repeated caerulein injection-induced CP murine models. (o) Representative images of H&E staining and tissue edema scoring of pancreatic tissues from repeated caerulein-challenged mice treated with vehicle (*n* = 7) or GW4064 (*n* = 7). Scale bars, 250 *μ*m. (p) Representative images of Sirius Red staining and evaluation of fibrosis of pancreatic tissues. Scale bars, 250 *μ*m. (q) Representative images of F4/80 immunostaining and number of F4/80^+^ cells/*μ*m^2^ of pancreatic tissues. Scale bars, 250 *μ*m. Data are presented as the mean ± SEM. ^∗^*P* < 0.5, ^∗∗∗^*P* < 0.001, and ^∗∗∗∗^*P* < 0.0001 via one-way ANOVA. ^#^*P* < 0.05, ^##^*P* < 0.01, and ^###^*P* < 0.001 via unpaired Student's *T* test. ns: not significant.

To further confirm whether FXR activation is protective in pancreatitis, we next treated murine models of caerulein-induced AP with the FXR agonist GW4064 (Figure 2(a)). As a result, GW4064 significantly reduced serum amylase and lipase level (Figure 2(b)) and alleviated pancreatic tissue edema and necrosis (Figures 2(c) and (d)) in caerulein-challenged FXR^f/f^ mice. GW4064 also alleviated neutrophil infiltration and acinar cell apoptosis in caerulein-induced pancreatitis (Figures 2(e) and 2(f)). Meanwhile, in caerulein-challenged FXR^acinar*Δ*/*Δ*^ mice, GW4064 treatment lost the beneficial effects on pancreatitis (Figures 2(g)–2(i)), indicating that pancreatic acinar FXR is required for the therapeutic effects of GW4064 on AP. To see whether FXR activation has therapeutic effects on other murine models of pancreatitis, we further used GW4064 to treat mouse models of L-arginine-induced AP (Figures 2(j)–2(m)) and repeated caerulein injection-induced CP (Figures 2(n)–2(q)). In results, GW4064 significantly reduced serum amylase and lipase levels (Figure 2(k)) and alleviated pancreas tissue edema and necrosis (Figures 2(l) and 2(m)) in mice treated with L-arginine. GW4064 also mitigated pancreas tissue edema, fibrosis, and chronic inflammation (Figures 2(o)–2(q)) in repeated caerulein injection-induced CP models. These data further indicate that FXR activation is beneficial in pancreatitis.

### 2.4. FXR Deletion Compromised the Viability and Stress Tolerance of Pancreatic Exocrine Organoids

**Figure 3:**
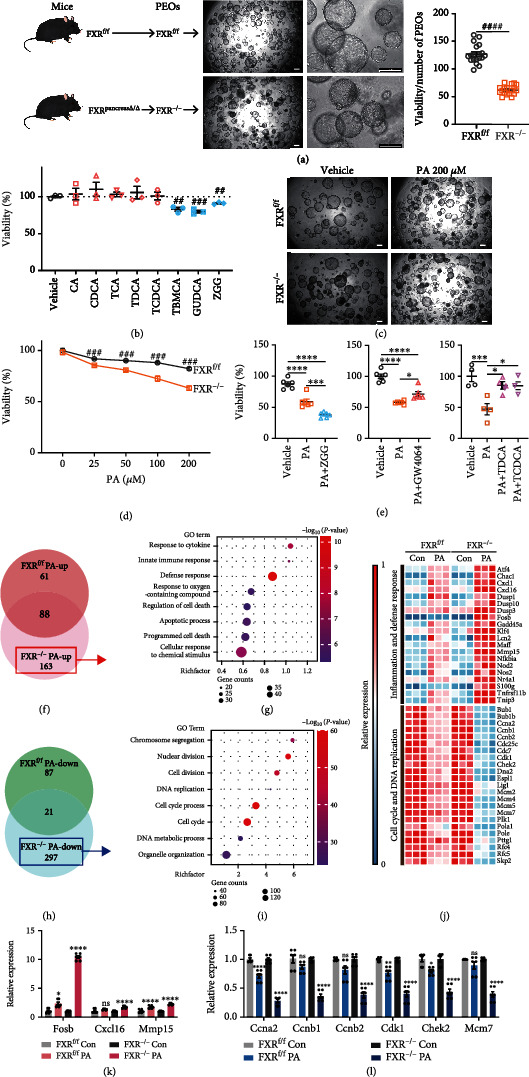
FXR deletion compromised the viability and stress tolerance of PEOs. (a) PEOs were initiated from mice and observed by microscopy. Representative images and the ratio of viability to number of FXR^f/f^ and FXR^−/−^ PEOs were performed. Scale bars, 200 *μ*m. (b) Viability of PEOs treated with 10 *μ*M CA, CDCA, TCA, TDCA, TCDCA, T-*β*MCA, GUDCA, and ZGG. (c) Representative microscopy images of FXR^f/f^ or FXR^−/−^ PEOs upon vehicle or PA treatment. Scale bars, 200 *μ*m. (d) Viability of FXR^f/f^ and FXR^−/−^ PEOs treated with PA at different concentrations. Viability was normalized to vehicle group values. (e) Viability of PEOs under PA challenge treated with ZGG, GW4064, TDCA, and TCDCA. (f) Venn analysis between upregulated genes in FXR^f/f^ and those in FXR^−/−^ PEOs upon PA stress. (g) GO enrichment of genes upregulated in FXR^−/−^ but not in FXR^f/f^ PEOs by PA treatment. (h) Venn analysis between downregulated genes in FXR^f/f^ and those in FXR^−/−^ PEOs upon PA stress. (i) GO enrichment of genes downregulated in FXR^−/−^ but not in FXR^f/f^ PEOs by PA treatment. (j) Heatmap of genes related to inflammation, defense response, cell cycle, and DNA replication changed by PA stress in FXR^f/f^ PEOs and showed more prominent changes in FXR^−/−^ PEOs than those in FXR^f/f^ PEOs. (k, l) RT-qPCR analysis of inflammation-related genes (Fosb, Cxcl16, and Mmp15) and cell cycle-related genes (Ccna2, Ccnb1, Ccnb2, Cdk1, Chek2, and Mcm7). ns: not significant. ^##^*P* < 0.01, ^###^*P* < 0.001, and ^####^*P* < 0.0001 via unpaired Student's *t* test. ^∗^*P* < 0.5, ^∗∗∗^*P* < 0.001, and ^∗∗∗∗^*P* < 0.0001 via one-way ANOVA.

As pancreatic exocrine organoids (PEOs) are excellent *in vitro* models for studying pancreatic exocrine functions [18], we next generated FXR^f/f^ PEOs from FXR^f/f^ mice and FXR^−/−^ PEOs from mice with FXR deletion in the whole pancreas (FXR^pancreas*Δ*/*Δ*^) (Figure 3(a) and Figure S2, a and b). FXR^−/−^ PEOs exhibited damaged organoid structures and a lower viability, indicating FXR is required for normal pancreatic organoids function (Figure 3(a)). We then treated the PEOs with different types of bile acids (BAs) as physiological FXR agonists and antagonists *in vitro* (Figure 3(b)). Upon treatment with the FXR antagonists tauro-*β*-muricholic acid (T-*β*MCA), glycoursodeoxycholic acid (GUDCA), and Z-guggulsterone (ZGG), the viability of PEOs was slightly reduced (Figure 3(b)).

To establish a model of pancreatic injury in PEOs, we tested the effects of several commonly used stress stimuli on wildtype (WT) PEOs (Figure S2c). Among these candidates, caerulein, tumor necrotic factor (TNF), asymmetric dimethylarginine (ADMA), and cortisone did not damage the PEOs, while palmitic acid (PA) and linoleic acid (LA) markedly impaired the viability and growth of PEOs (Figure S2, c and d). Moreover, we performed RNA-seq analysis on PA-treated PEOs and found that genes upregulated upon PA treatment in FXR^f/f^ PEOs were primarily related to the NLRP3 inflammasome, mitophagy, and noncanonical NF-*κ*B signaling (Figure S3, a-f), which are well-recognized molecular cascades implicated in the pathogenesis of AP. Thus, we considered PA-induced PEOs damage model as an appropriate in vitro model of pancreatitis.

Next, we compared the responses of FXR^f/f^ and FXR^−/−^ PEOs to PA and found that FXR^−/−^ PEOs exhibited lower tolerance to PA stress (Figures 3(c) and 3(d)). The PA-induced reduction in PEO viability was attenuated by the FXR agonists GW4064, TDCA, and TCDCA, while aggravated by the FXR antagonist ZGG (Figure 3(e)). Furthermore, we analyzed genes that exhibited differential expression only in FXR^−/−^ PEOs but not in FXR^f/f^ PEOs under PA stress. The upregulated genes were mainly enriched in inflammatory and defense responses (Figures 3(f) and 3(g)), while the downregulated genes were involved in DNA replication and cell cycle (Figures 3(h) and 3(i)). In addition, some genes related to inflammation, defense response, cell cycle, and DNA replication in FXR^f/f^ PEOs under PA-induced stress exhibited more prominent expression changes in FXR^−/−^ PEOs, which was also validated via quantitative real time PCR (RT-qPCR) (Figures 3(j)–3(l)). These results indicated that FXR ablation caused more severe inflammation and impaired renewal ability in PEOs.

### 2.5. Osgin1 Was Transcriptionally Upregulated by FXR Activation in PEOs

**Figure 4:**
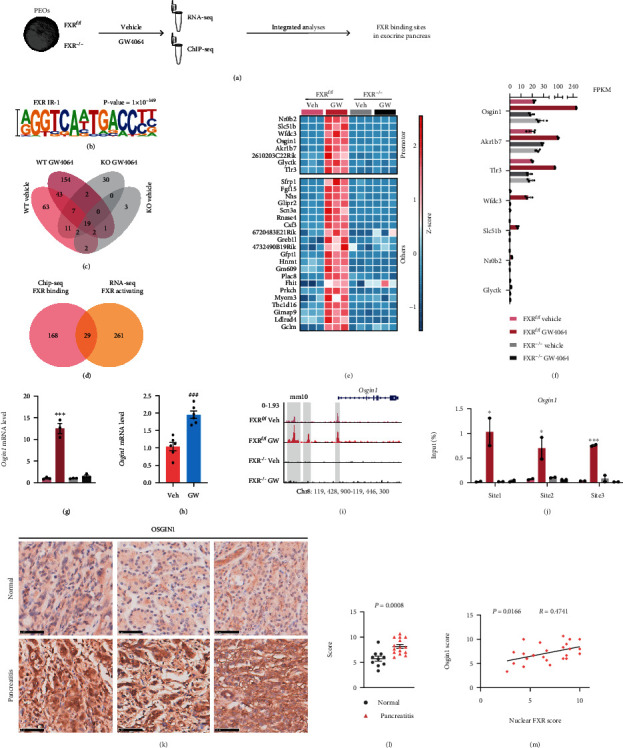
*Osgin1* was transcriptionally upregulated by FXR activation in PEOs and OSGIN1 level was elevated in human pancreatitis tissues. (a) Study design. (b) The top represented de novo motifs in FXR in GW4064-treated FXR^f/f^ PEOs. (c) Venn analysis among peaks called from the four groups. (d) Venn analysis between genes with FXR binding from ChIP-seq analysis and genes upregulated by FXR activation from RNA-seq analysis. (e) Heatmap of genes transcriptionally activated by FXR. (f) FPKM values of genes, which were bound by FXR at their transcription start site (TSS) and promoter regions. (g) qPCR analysis of *Osgin1* expression in FXR^f/f^ or FXR^−/−^ PEOs treated with or without GW4064. (h) qPCR analysis of *Osgin1* expression in pancreatic tissues of mice treated with or without GW4064. (i) FXR binding sites in the *Osgin1* upstream sequence are highlighted. (j) ChIP-qPCR validation of FXR binding sites in the *Osgin1* upstream sequence. (k) Representative images of OSGIN1 IHC staining in three pancreatitis tissues and three normal tissues. Scale bars, 50 *μ*m. (l) Scoring for the OSGIN1 levels in human pancreatic tissues (normal tissues, *n* = 10; pancreatitis tissues, *n* = 15). (m) Correlation between nuclear FXR score and OSGIN1 score. Data are presented as the mean ± SEM. ns: not significant. ^∗∗^*P* < 0.01 and ^∗∗∗^*P* < 0.001 via one-way ANOVA compared with WT vehicle group.

To identify the downstream signaling of FXR that majorly functions in exocrine pancreatic cells, we subjected PEOs with or without GW4064 treatment to RNA-seq (Figure S4) and ChIP-seq (Figure 4(a) and S5). Upon activation, FXR is bound to the promoter of *Shp* and *Slc51b*, enhancing their mRNA expression (Figure S5, c-f), which is consistent with previous reports [19] in liver and intestine. Motif analysis revealed that the binding motif of FXR in PEOs was an inverted repeat spaced by one nucleotide (IR-1) (Figure 4(b)). Venn analysis identified 29 genes that were transcriptionally upregulated by FXR activation in PEOs (Figures 4(c) and 4(d)). Among these, FXR is bound to the transcription start site (TSS) and promoter regions of eight genes (Figure 4(e)). Considering that the FPKM value of *Osgin1* was the highest (Figure 4(f)), we hypothesized that *Osgin1* is a major target of FXR within cells of the exocrine pancreas. Indeed, as shown in Figure 4(g), the mRNA levels of *Osgin1* increased ~13-fold in GW4064-treated WT PEOs, while this effect was abolished in GW4064-treated FXR KO PEOs. In vivo, the mRNA levels of *Osgin1* increased ~2-fold after GW4064 treatment (Figure 4(h)). Next, we performed ChIP-qPCR and found that FXR is bound to 6592-6774, 6331-6518, and 63-243 upstream of the *Osgin1* TSS (Figures 4(i) and 4(j)). By analyzing the public GEO dataset (GSE133700), we also found that *Osgin1* could harbor the binding sites of four FXR isoforms in liver organoids (Figure S5g) [20].

### 2.6. Osgin1 Mediated the Protective Effects of FXR on Pancreatitis

We further assessed the clinical significance of OSGIN1 in pancreatitis by IHC and found that OSGIN1 level in human pancreatitis tissues was significantly elevated compared to that in normal pancreatic tissues (Figures 4(k) and 4(l)). Additionally, the OSGIN1 level was significantly correlated with nuclear FXR level (Figure 4(m)).

**Figure 5:**
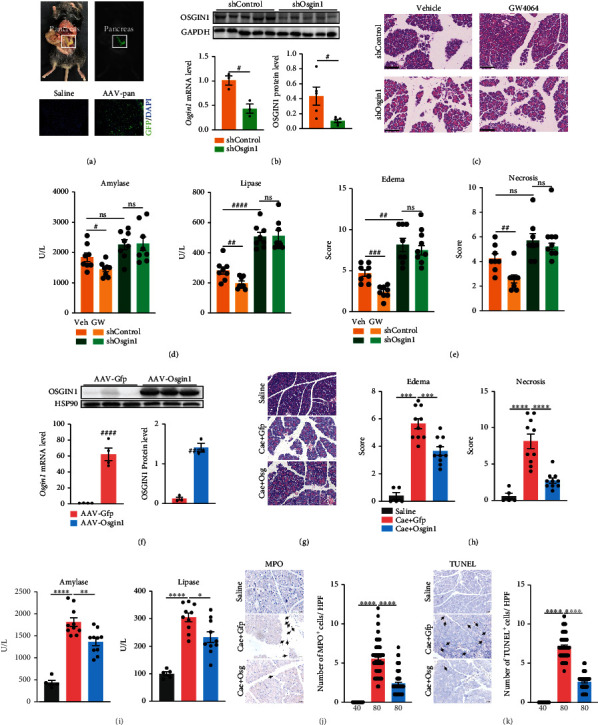
*Osgin1* mediated the protective effects of FXR activation on pancreatitis. (a) Representative in vivo fluorescent imaging of GFP in pancreas of mice after AAV-pan transduction and frozen slices of GFP in pancreatic tissue from mice with AAV-pan transduction. Scale bars, 100 *μ*m. (b) Confirmation of *Osgin1* knockdown by RT-qPCR and immunoblots analysis. (c) Representative images of H&E staining in pancreatic tissues. Scale bars, 100 *μ*m. (d) Serum amylase and lipase levels of caerulein challenged mice in four groups (shControl + vehicle, *n* = 8; shControl + GW4064, *n* = 8; sh*Osgin*1 + vehicle, *n* = 8; sh*Osgin*1 + GW4064, *n* = 8). (e) Scoring for tissue edema and necrosis. (f) Confirmation of *Osgin1* overexpression by RT-qPCR and immunoblots analysis. (g) Representative images of H&E staining in pancreatic tissues. Scale bars, 20 *μ*m. (h) Scoring for tissue edema and necrosis in three groups (saline, *n* = 5; caerulein + AAV − Gfp, *n* = 10; caerulein + AAV − Osgin1, *n* = 10). (i) Serum amylase and lipase levels of mice in four groups. (j) Representative images of MPO immunostaining and its statistics plot. Scale bars, 20 *μ*m. (k) Representative images of TUNEL immunostaining and its statistics plot. Scale bars, 20 *μ*m. Five HPFs per individual. The number of HPFs used for the summarized plots of MPO and TUNEL immunostaining is indicated. Data represent the mean ± SEM. ^#^*P* < 0.05, ^##^*P* < 0.01, ^###^*P* < 0.001, and ^####^*P* < 0.0001 via unpaired Student's *t* test. ^∗^*P* < 0.5, ^∗∗^*P* < 0.01, and ^∗∗∗∗^*P* < 0.0001 via one-way ANOVA. ns: not significant.

To investigate the functional role of *Osgin1* in vivo, we manipulated *Osgin1* in the pancreatic tissues utilizing AAV-pan, in which the AAV2 genomes were encapsulated in the Y447F and Y773F mutant AAV8 capsids to generate a viral vector with high transduction efficiency in pancreatic acinar cells [21]. We infected mice with AAV-pan with a cytomegalovirus (CMV) promoter driving green fluorescent protein (AAV-pan-CMV-GFP) expression and found a high infection efficiency of AAV-pan in pancreatic tissues using an imaging system for live animals and fluorescence microscopy of frozen slices (Figure 5(a)). We then infected mice with AAV-sh*Osgin1* or AAV-shControl by intraperitoneal injection (i.p.). After 2 weeks, the mice were subjected to AP modeling and GW4064 treatment. RT-qPCR and immunoblots analyses showed that AAV-sh*Osgin1* successfully suppressed the mRNA and protein levels of *Osgin1* (Figure 5(b)). In mice with *Osgin1* knockdown, no difference was observed in the serum amylase and lipase levels as well as tissue edema and necrosis between the vehicle and GW4064 group (Figures 5(c)–5(e)), suggesting that the therapeutic effects of GW4064 on pancreatitis were mediated by the promotion of *Osgin1* expression.

Meanwhile, we found that *Osgin1* knockdown exacerbated pancreatitis. The serum lipase level and tissue edema were significantly increased by *Osgin1* knockdown (Figures 5(c)–5(e)). Therefore, we further overexpressed OSGIN1 in the mouse pancreas using AAV-pan to investigate whether Osgin1 could protect against pancreatitis. AAV-pan with a cytomegalovirus (CMV) promoter driving *Osgin1* expression (AAV-pan-CMV-*Osign1*) was constructed, and AAV-pan with a CMV promoter driving green fluorescent protein (AAV-pan-CMV-GFP) expression served as a control. Overexpression of *Osgin1* in the pancreas was confirmed through RT-qPCR and western blotting (Figure 5(f)). Two weeks after the injection of AAV-*Osgin1* or AAV-GFP, mice were challenged with caerulein for seven hours. *Osgin1* overexpression in the pancreas mitigated the elevation of serum amylase and lipase levels, tissue edema and necrosis, inflammation infiltration, and acinar cell apoptosis in mice with caerulein challenge (Figures 5(g)–5(k)), which was consistent with the results of GW4064 administration.

### 2.7. FXR Activation Restores Efficient Autophagy in Pancreatitis by Enhancing Osgin1 Expression

**Figure 6:**
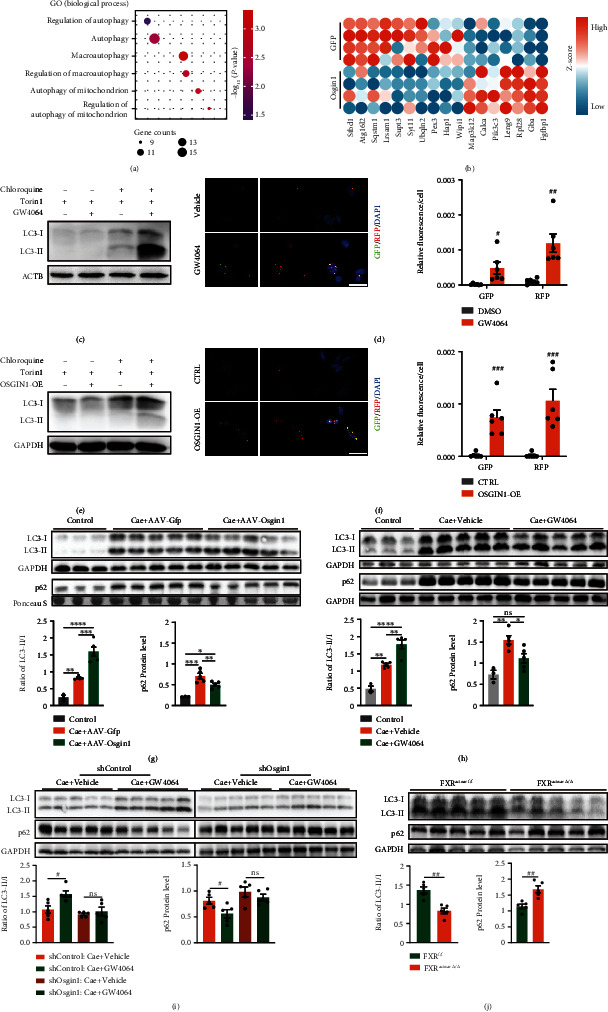
FXR enhanced restored efficient autophagy via enhancing *Osgin1* expression in pancreatitis. (a) GO enrichment of genes changed by *Osgin1*-overexpression. Plot represented autophagy-related biological process. (b) Heatmap of autophagy-related genes changed by *Osgin1*-overexpression. (c) Representative immunoblot images of LC3I/II in SW1990 cells changed by GW4064 treatment. (d) Representative images and statistics plot of GFP and RFP puncta in SW1990 cells changed by GW4064 treatment. (e) Representative immunoblots images of LC3I/II in SW1990 cells changed by OSGIN1 overexpression (OSGIN1-OE). (f) Representative images and statistics plot of GFP and RFP puncta in SW1990 cells changed by OSGIN1-OE. (g) Representative western blot images and summarized plot of p62 protein levels as well as the ratio of LC3-II to LC3-I in pancreatic tissues from mice treated with saline (*n* = 3), caerulein + AAV − pan − CMV − Gfp (*n* = 5), or caerulein + AAV − pan − CMV − *Osgin*1 (*n* = 5). (h) Representative western blot images and summarized plot of p62 protein levels as well as the ratio of LC3-II to LC3-I in pancreatic tissues from mice treated with saline + vehicle (*n* = 3), caerulein + vehicle (*n* = 5), or caerulein + GW4064 (*n* = 5). (i) Representative western blot images and summarized plot of p62 protein levels as well as the ratio of LC3-II to LC3-I in pancreatic tissues from caerulein challenged mice treated with AAV − shControl + vehicle (*n* = 5), AAV − shControl + GW4064 (*n* = 5), AAV − sh*Osgin*1 + vehicle (*n* = 5), and AAV − sh*Osgin*1 + GW4064 (*n* = 5). (j) Representative western blot images and summarized plot of p62 protein levels as well as the ratio of LC3-II to LC3-I in pancreatic tissues from FXR^f/f^ (*n* = 5) and FXR^acinar*Δ*/*Δ*^ (*n* = 5) mice treated with caerulein. Data are presented as the mean ± SEM. ns: not significant. ^∗^*P* < 0.5, ^∗∗^*P* < 0.01, ^∗∗∗^*P* < 0.001, and ^∗∗∗∗^*P* < 0.0001 via one-way ANOVA. ^#^*P* < 0.01 and ^##^*P* < 0.01 via unpaired Student's *t* test.

Previous studies showed that OSGIN1 is an important regulator of autophagy [22–24]. In smoking-induced stress in human airway epithelium, OSGIN1 induced the formation of autophagosomes to enhance autophagy [22]. Autophagy is critical for pancreatic injury repair, eliminating injured and useless cell components and recycling their constituents [25]. To look into the downstream signaling of OSGIN1, we next conducted RNA-seq on pancreatic tissues with OSGIN1 overexpression (OSGIN1-OE) and performed GO enrichment analyses. It revealed that genes altered by OSGIN1-OE were significantly enriched in autophagy-related biological processes (Figures 6(a) and 6(b)). We treated pancreatic cells (SW1990) with GW4064 in presence of Torin1, which inhibits the mTORC1/2 complex to induce autophagy [26]. The results showed that in the presence of chloroquine, GW4064 treatment significantly increased levels of LC3-II (Figure 6(c)). Furthermore, we constructed an adenovirus vector with an Ubi promoter driving the expression of a dual-fluorescence fusion protein, stubRFP-sensGFP-LC3B. This protein could reflect the level of autophagic flux in cells, autophagosomes that are not fused with lysosomes present yellow signals (overlapping of red and green fluorescence), whereas the red signal corresponds to autolysosomes. Here, SW1990 cells were transfected with the stubRFP-sensGFP-LC3B adenovirus (Ad-Ubi-stubRFP-sensGFP-LC3B) followed by Torin1 and GW4064 treatment. The results showed that the yellow and red puncta were both increased upon GW4064 treatment (Figure 6(d)), suggesting that autophagosomes and autolysosomes were significantly increased by FXR activation. The RT-qPCR analysis showed that the *Osgin1* mRNA level was also elevated by GW4064 treatment in SW1990 cells (Figure S6a). We then overexpressed OSGIN1 in SW1990 cells (Figure S6a) and detected the alterations of autophagic flux. The results were similar to those with GW4064 treatment (Figures 6(e) and 6(f)). Furthermore, we overexpressed OSGIN1 in both 293T and SW1990 cells and conducted immunoprecipitation followed by mass spectrometry (IP-MS). The Venn analysis identified 48 proteins that interacted with OSGIN1 (Figure S6b). The Reactome pathway enrichment revealed that OSGIN1 was interacted with OSGIN1 interacted with most of the critical components of the ATP-dependent chaperonin complex TRiC (also called CCT) (Figure S6, b and c), which is reportedly involved in the autophagic flux [27, 28], further supporting the regulation of autophagy by OSGIN1.

Next, we investigated the effects of FXR-OSGIN1 signaling on autophagy in pancreatic tissues, and we assessed LC3-I, LC3-II, and p62 protein levels *in vivo* using immunoblot. In caerulein-induced mouse models of pancreatitis, OSGIN1 overexpression significantly reduced the accumulation of p62 and increased the ratio of LC3-II to LC3-I (Figure 6(g)), which was consistent with the results in GW4064 treatment (Figure 6(h)). We observed the autophagic vesicles in pancreatic tissues utilizing transmission electron microscopy (TEM). It showed that autophagic vesicles were dramatically accumulated in pancreatitis tissues and were significantly reduced by OSGIN1-OE or GW4064 treatment (Figure S6d). Moreover, the autophagy promoting effect of GW4064 was almost totally abolished by *Osgin1*-knockdown (Figure 6(i)), and FXR deletion in pancreatic acinar cells led to a more severe impairment of autophagy (Figure 6(j) and S6d). These findings indicated that FXR activation restored efficient autophagy by promoting the expression of *Osgin1*.

## 3. Discussion

In this study, we find that the key bile acid receptor FXR in pancreatic acinar cells is activated in pancreatic tissues and required for the protective effects against pancreatitis. The FXR agonist GW4064 exhibited excellent therapeutic effects on pancreatitis; it could directly activate pancreatic acinar FXR to restore efficient autophagy mediated by enhanced *Osgin1* expression, thus mitigate the severity of pancreatitis.

Previous reports showed that global FXR knockout did not have significant effects on experimental AP [29]. The impact of global FXR deletion on the whole organism, especially the liver and intestine, is considerably complex, and specific FXR function in pancreatitis may not be easily observed. Tissue-specific deletion of FXR is a better way to investigate its unique roles in pancreas. To this end, we generated mutant mice of pancreatic acinar cell-specific deletion of FXR (FXR^acinar*Δ*/*Δ*^), serving as an appropriate in vivo model to investigate the functions of FXR in pancreatitis. Zhou et al. previously considered FXR activation in CP as a deleterious pathological alteration based on their finding that nuclear FXR expression was positively correlated with the severity of CP in human patients [30]. However, without detailed mechanistic study *in vitro* and *in vivo*, their conclusion ruled out the possibility that FXR upregulation was compensatory and beneficial in pancreatitis. Indeed, here, we found that acinar cell-specific deletion of FXR in mice caused more severe pancreatitis both in acute and chronic pancreatitis models, indicating that FXR played a protective role against pancreatitis.

Impaired autophagy has been shown to be an important pathological event in AP. Inhibition of autophagy through genetic or pharmacological approaches led to more severe pancreatitis in mice, while restoration of efficient autophagy via disaccharide trehalose mitigated the disease [31, 32]. Therefore, restoration of efficient autophagy in the acinar cells could be beneficial to alleviate pancreatitis [33]. Indeed, we provided sufficient evidences in this study showing that FXR actually stimulated autophagy in the pathogenesis of pancreatitis. This is very different from the situation in the liver, where FXR is an autophagy suppressor in the fasting condition [34, 35]. As shown by our results, FXR agonist GW4064 and overexpression of its downstream OSGIN1 stimulated autophagic flux in pancreatic cell lines. In vivo, deletion of FXR resulted in a more severe impairment of autophagic flux, whereas the FXR agonists GW4064 significantly stimulated the autophagic flux and reduced the accumulation of autophagic vesicles in the pancreatic tissues of mice with pancreatitis. These results demonstrate that FXR is an autophagic regulator that promotes autophagic flux in pancreatic cells.

Of note, only 11% of FXR-binding sites in the liver and intestine are overlapped [36], suggesting that FXR may exert specific molecular functions in different organs. It is possible that FXR plays unique functions in the pancreas or at least via regulating a different set of downstream genes. In the liver, FXR inhibits the expression of autophagic genes, such as Map1lc3a, Map1lc3b, Atg2a, Atg2b, Atg7, and Atg10 through directly binding to their promotor or disrupting the CREB/CRTC2 complex [34, 35]. In our ChIP-seq and RNA-seq data of PEOs, we did not observe binding sites or transcriptional regulation of FXR on autophagy genes, suggesting that, in the exocrine pancreas, FXR regulates autophagy not by directly transcriptional promote or inhibit autophagic gene expression but by a unique way.

It is already known that all the four FXR isoforms bind to the promoter of *Osgin1* in the liver organoids [20], but no literatures report the biological or clinical significance of this transcriptional regulation. Here, our study gives FXR-OSGIN1 axis important biological and pathological implications for the first time. We find that OSGIN1 is elevated in human pancreatitis tissues and positively correlated with nuclear FXR expression. OSGIN1 is transcriptionally activated by direct binding of FXR to its promotor in pancreatic exocrine cells and mediates the protective effects of FXR on pancreatitis in vivo. OSGIN1 has been recognized as a regulator of autophagy in several studies. In human airway basal cells, overexpressed OSGIN1 upregulated and colocalized with LC3-II [22]. Overexpression of OSGIN1 also increased LC3-II and decreased p62 protein levels in MCF-7 cells, while knockdown impaired autophagy in HeLa cells [24]. lncRNAs UCA1 and METTL3 were found to regulate autophagy by targeting *Osgin1* to affect cell death [23, 37]. Consistent with the findings with these studies, overexpression of OSGIN1 stimulated autophagic flux in pancreatitis tissues and cell lines, which was similar to the effect of GW4064 treatment. As illustrated in Figure S6, b and c, OSGIN1 interacted with most of the critical components of CCT. CCT plays important roles in maintaining efficient autophagic flux. A loss of CCT function leads to the inability to process aggregated proteins and inhibition of autophagy [27]. Recently, it was reported that the chaperonin subunit CCT2 is an aggrephagy receptor, regulating the clearance of aggregation-prone proteins [28]. CCT also folds both mLST8 and Raptor to play a key role in mTORC assembly and signaling [38]. We consider the interaction between OSGIN1 and CCT is likely to be the intrinsic mechanism by which OSGIN1 regulates the autophagy. In our future study, we will focus on this interaction and investigate whether it is involved in the regulation of autophagy by OSGIN1.

Overall, our results showed that the FXR-OSGIN1 axis plays an important role in the regulation of autophagy and protects against pancreatitis in the exocrine pancreas. Activation of pancreatic acinar FXR restores efficient autophagy by promoting OSGIN1 expression to ameliorate pancreatitis. Our results highlight the FXR-OSGIN1 axis as a promising pharmacological target for treating pancreatitis.

## 4. Materials and Methods

### 4.1. Experimental Design

We assessed FXR and OSGIN1 protein levels in human pancreatitis tissues through IHC to demonstrate the clinical significance of our study. We specifically depleted pancreatic acinar FXR in mice and treated different mouse models of pancreatitis with FXR agonists GW4064 to explore the effects of FXR activation in pancreatitis. PEOs with or without FXR deletion were used as in vitro models. RNA-seq and ChIP-seq analyses of PEOs were conducted to identify factors acting downstream of FXR in the exocrine pancreas. We utilized AAV-pan to knockdown or overexpress Osgin1 specifically in the pancreas of mice in order to investigate the role of Osgin1 in AP. Immunoblots and Ad-LC3B-dual reporter of autophagy flux were used to evaluate autophagy in vitro. Immunoblots and TEM were conducted to evaluate autophagy in vivo.

### 4.2. Human Samples

All patients were from The First Affiliated Hospital of Zhengzhou University, Zhengzhou, China. All patients and/or legal guardians signed the informed consent documentation prior to experiments. Normal pancreatic tissues (*n* = 10) were obtained from patients with pancreatic cancer in the pancreatectomy. Pancreatitis tissues (*n* = 15) were obtained from patients diagnosed with pancreatitis. Six of them were obtained through biopsy and nine were from pancreatectomy. Detailed information of these patients was collected in Table S1. All research procedures on human samples were conducted with approval of the Ethics Committee of the First Affiliated Hospital of Zhengzhou University and in line with the Declaration of Helsinki.

### 4.3. Mice

Mice of the C57BL/6J strain (Department of Laboratory Animal Science in Fudan University) were maintained at room temperature (22-25°C) and with free access to food and water with 12 h light/dark cycle in. FXR^f/f^ mice with a C57BL/6J background were provided by Prof. Changtao Jiang. Mist1^CreERT2^ mice with a C57BL/6J background were purchased from The Jackson Laboratory. PDX1^cre^ mice with a C57BL/6J background were purchased from GemPharmatech. Pancreatic acinar deletion of FXR (FXR^acinar*Δ*/*Δ*^) was induced in Mist1^CreERT2^; FXR^f/f^ mice via intraperitoneal injection of tamoxifen (100 mg/kg) for 5 days. The FXR in whole pancreas of PDX1^Cre^; FXR^f/f^ mice was depleted from birth. The growth and development of FXR^acinar*Δ*/*Δ*^ and FXR^pancreas*Δ*/*Δ*^ mice were normal, and the body weight was comparable among FXR^acinar*Δ*/*Δ*^ FXR^pancreas*Δ*/*Δ*^, and FXR^f/f^ mice of the same sex and age.

All animal studies were performed according to protocols approved by the Animal Ethics Committee of the Fudan University School of Basic Medical Sciences. All mice were used after 1- or 2-weeks acclimatization after importing into the facility. Before each experiment, mice of the same group were placed in the same appropriately sized cage and equilibrated for a week. During equilibration, mice in control group and treated group were kept in the same condition. The number of mice was decided with reference to Guideline for the Care and Use of Laboratory Animals in China. Humane endpoints were defined as any obvious disease-related symptoms in mice. We established humane endpoints, but no mice were humanely sacrificed in this study.

### 4.4. Pancreatitis Modeling

Pancreatitis modeling was performed as described in the figures. Briefly, to establish caerulein-induced pancreatitis models, mice were intraperitoneally injected with 50 *μ*g/kg caerulein 6 hourly and were sacrificed 1 hour after the last caerulein injection. To establish caerulein-induced CP models, the above procedure was repeated 6 times in two weeks, and mice were sacrificed 1 hour after the last injection. To establish L-arginine-induced AP models, mice were intraperitoneally injected with 4 g/kg L-arginine (10%) twice with 1 hour interval and were sacrificed 6 hours after the first injection.

### 4.5. Histological Evaluation

IHC staining of FXR and OSGIN1 was analyzed by histological scoring. Scoring criteria was divided into two parts: the intensity of protein expression (score = 0, none; score = 1, mild; score = 2, moderate; score = 3, severe) and the quantity of protein expression (score = 0, none; score = 1, 1% ~ 25%; score = 2, 26% ~ 50%; score = 3, 51% ~ 75%; and score = 4, 76% ~ 100%). Pancreatic injury of mice was evaluated by pancreatic edema, necrosis. Scoring criteria for quantity (the estimated proportion of edema and necrosis) were as follows: score = 0, none; score = 1, 1% ~ 25%; score = 2, 26% ~ 50%; score = 3, 51% ~ 75%; and score = 4, 76% ~ 100%. Scoring criteria for intensity of pancreatic edema were as follows: score = 0, none; score = 1, acinar swelling; score = 2, acinar swelling and interlobular interstitial separation; and score = 3, severe interlobular interstitial separation. Scoring criteria for intensity of pancreatic necrosis were as follows: score = 0, none; score = 1, mild spotty necrosis; score = 2, severe spotty necrosis; and score = 3, diffuse spotty necrosis with lobular loss. Each sample was evaluated in a blinded manner by three senior pathologists. Final score of each sample was the average of three scores. The quantity and intensity scores were then multiplied to obtain a total score, which could range from 0 to 12. Inflammatory infiltration in AP was evaluated by the number of MPO positive cells per high power field. For each individual, we obtained one tissue section and performed MPO immunochemistry staining on it. We choose 5-10 high power fields to count the MPO positive cells in each tissue section. Cell apoptosis was evaluated by TdT-mediated dUTP nick end labeling (TUNEL) staining. We calculated the number of TUNEL positive cells per high power field. We also observed 5-10 high power fields of one section from one sample. Every point in column chart of inflammation and apoptosis evaluation performed the number of MPO or TUNEL positive cells in one high power field. Tissue fibrosis and inflammation infiltration in CP models were evaluated by Sirius Red staining and F4/80 immunostaining followed by analyses using Visiopharm (Hamamatsu). The area of total tissues and fibrotic tissues as well as the number of F4/80+ cells was calculated, respectively. We calculated the fibrotic percentages and number of F4/80+ cells/*μ*m^2^ to represent the fibrotic and inflammatory degree of each individual.

### 4.6. Pancreatic Exocrine Organoid (PEO) Initiation and Culturing

WT PEOs were initiated from FXR^f/f^ mice, and FXR KO PEOs were established from FXR^f/f^; PDX1^cre^ mice. Mice were sacrificed after anesthesia with 3% isoflurane. After cutting up, pancreatic tissues from mice were digested five times using a tissue dissociation cocktail containing 3.75 ml collagenase IV (1 mg/ml), 3.75 ml dispase (1 U/ml), 300 *μ*l DNase I solution (1 mg/ml), and 22.2 ml DMEM/F-12 with 5 mM HEPES. The digested liquid was filtered through a reversable strainer to preserve the ductal fragments and remove other cells. Remaining ductal fragments on the filter were collected and suspended in Matrigel. A 50 *μ*l suspension was added to the wells of a 24-well plate to form a dome. After 10 min of incubation in a 37°C incubator with 5% CO_2_, PEOs were cultured in 750 *μ*l commercial culture medium per well (PancreaCult™ Organoid Growth Medium). PEOs were passaged–5-6 days after establishment. Briefly, PEOs were broken into small pieces using a pipette in Advanced DMEM/F-12. The diameter of fragments was limited to less than 70 *μ*m using a strainer. The fragments were collected, mixed with Matrigel, and plated on cell culture plates. PEOs were cultured in commercial culture medium and passaged every 3-6 days.

### 4.7. Viability Assays of PEOs and the Area Calculation

The grown PEOs were broken into small pieces in Advanced DMEM/F-12 using a pipette. The diameter of fragments was also limited to less than 70 *μ*m using a strainer. Fragments were collected and suspended in Matrigel. To ensure that the organoids were evenly distributed, the suspension was pipetted up and down 5-8 times and immediately added into the wells of a 96-well plate (10 *μ*l per well). PEOs were cultured in 100 *μ*l culture medium per well for 2-3 days. To explore the effect of different substances on PEOs, the culture medium was removed carefully, and new culture medium with different content was added without touching the PEO dome. The viability of organoids was evaluated using the CellTiter-Glo® Luminescent Cell Viability Assay (Promega). Image J (Version 1.53) [39] was used to calculate the area of PEOs. Briefly, we set scales according to the ruler in images, drew the outline of PEOs along the edges, and measured the area.

### 4.8. RNA Extraction and Real Time-Quantitative Polymerase Chain Reaction (RT-qPCR)

TRIZOL reagent was used to extract total RNA. For RT-qPCR, reverse transcription process was performed to generate cDNA using HiScript III RT SuperMix for qPCR (+gDNA wiper). RT-qPCR was conducted through SYBR Green PCR kit, with 36b4 and *β*-actin being normalization/internal controls. All primers employed for such gene expression investigations are illustrated within Table S2.

### 4.9. RNA-Sequencing (RNA-Seq)

We used 1 *μ*g total RNA to construct cDNA library based on TrueLib mRNA Library Prep Kit for Illumina (ExCell Bio). The cDNA libraries were sequenced on an Illumina Hiseq (Illumina) by the Annoroad Company (Beijing, China). RNA-seq data were processed as described previously [40, 41]. The mRNA abundance was expressed in FPKM (fragments per kilobase of exon per million reads mapped). Primary FPKM data was processed by R (Version 4.1.0). Normalization, principal component analysis (PCA), and clustering analysis were performed as quality control. Student's *t* test, Venn analysis, gene oncology (GO) enrichment analysis, and Kyoto Encyclopedia of Genes and Genomes (KEGG) pathway enrichment analysis were all processed on R. Gene set enrichment analysis (GSEA) was computed on GSEA v4.2.0 Mac App [42, 43]. Heatmap was generated through pheatmap package on R Studio.

### 4.10. Chromatin Immunoprecipitation (ChIP) Followed by Sequencing or qPCR

FXR^f/f^ or FXR^−/−^ PEOs (10 domes/well) planted in 6-well plates was treated with or without 10 *μ*M GW4064 overnight and was crosslinked by 1% formaldehyde at room temperature for 10 minutes and quenched by 0.125 M glycine. Subsequent ChIP assays were conducted as described previously [44]. In brief, the crosslinked PEOs were suspended in ChIP lysis buffer (50 mM HEPES pH 7.5, 0.1% SDS, 0.1% sodium deoxycholate, 1% Triton X-100, 500 mM NaCl, 1 mM EDTA, and freshly added protease inhibitor cocktails) and sonicated to generate the sheared chromatin (200-300 bp). Anti-FXR antibody was then added in (dilution: 1 : 50) to incubate with the sheared chromatin overnight at 4°C. After immobilization on prewashed rProtein A/G Magpoly beads, the protein-DNA complexes were washed three times with ChIP lysis buffer, twice with low-salt buffer (10 mM Tris-HCl pH 8.0, 0.5% sodium deoxycholate, 0.5% NP-40, 250 mM LiCl, 1 mM EDTA, and freshly added protease inhibitor cocktails), and once with TE buffer (10 mM Tris-HCl pH 8.0 and 1 mM EDTA). Elution and reverse crosslinking were carried out in the elution buffer (50 mM Tris-HCl pH 8.0, 1% SDS, 10 mM EDTA, freshly added RNase A and proteinase K) at 65°C for 4 hours. After digestion for 1 hour at 55°C, DNA samples were purified using a PCR purification kit. Primers for ChIP-qPCR were in Supplemental Table 3. Library preparation was performed by VAHTS Universal DNA Library Prep Kit and sequenced on NovaSeq 6000 by the Annoroad Company (Beijing, China). The FASTQ data were trimmed by Trim Galore (v0.4.4_dev) and mapped to the mouse genome (mm10 version) using Bowtie2 (v2.3.4.1) [45]; then, peaks were identified by macs2 (v2.1.2) [46]. Genome Coverage bigwig files, signalplots, and heatmaps were generated by deeptools (v3.3.0) [47]. Bigwig files were visualized by IGV(v2.1.3) [48]. ChIP-seq between FXR^f/f^ and FXR^−/−^ were normalized by total reads.

### 4.11. Cell Culture and Transfection

The human SW1990 cells were cultured in 5% CO2 atmosphere at 37°C in Leibovitz's L-15 medium supplemented with 10% fetal bovine serum and 100 units/ml penicillin/streptomycin. For transfection, 2.4^∗^10^6^ cells were collected by digestion and centrifuge and then added into 300 *μ*l medium with 25 *μ*l R buffer. We conducted electron transfection in SW1990 cells to overexpress Osgin1 (pLV3-CMV-OSGIN1(human)-3^∗^FLAG-Puro) using MPK5000 Neon™ Transfection System (1150 V, 30 ms, 2 pulses). Cells were then seeded in plates to be processed as described.

### 4.12. Osgin1 Manipulation in Pancreas of Mice

The viral tools were all packaged by BrainVTA (Wuhan) Co., Ltd. AAV viral vectors were constructed according to Chen et al. [21]. AAV2 genomes were encapsulated in the Y447F and Y773F mutant AAV8 capsids to generate a viral vector with high transduction efficiency for the pancreatic acinar cells (AAV-pan). For *Osgin1* knockdown, AAV-pan with a U6 promoter driving sh*Osgin1* (GATGCTATACCCTGAGTACCA) and cytomegalovirus (CMV) promoter driving mCherry (AAV-pan-U6-sh*Osign1*-CMV-mCherry) was constructed, and AAV-pan with a U6 promoter driving scramble (CCTAAGGTTAAGTCGCCCTCG) and CMV promoter driving mCherry protein (AAV-pan-U6-shControl-CMV-mCherry) expression served as a control. For *Osgin1* overexpression, AAV-pan with a cytomegalovirus (CMV) promoter driving *Osgin1* expression (AAV-pan-CMV-Osign1) was conducted and AAV-pan with CMV promoter driving green fluorescent protein (AAV-pan-CMV-GFP) served as a control. AAV was injected (1^∗^10^11^ vg per mouse) intraperitoneally two weeks before modeling. Successful transduction for every mouse was confirmed by in vivo fluorescence imaging after sacrifice.

### 4.13. Immunoblot

Samples were snap-frozen in liquid nitrogen immediately upon harvesting. RIPA buffer with freshly added protease inhibitor cocktail, and phenylmethylsulfonyl fluoride was used to lyse frozen-samples and extract proteins. We loaded equal amounts of protein (30–50 *μ*g) on an SDS-polyacrylamide gel to separate them. Proteins were then electrotransferred onto PVDF membranes (Millipore), which were then blocked in 5% skim milk in Tris-buffered saline containing Tween 20 for 1 h at room temperature. The membranes were probed with primary antibodies overnight at 4°C and incubated at room temperature for 1 h with an appropriate secondary antibody conjugated to horseradish peroxidase. The protein signals were visualized with an ECL Western Blotting kit (Tanon). ImageJ was used to calculate protein relative expression by plot every gel lane. All protein expression was normalized by Ponceau S or loading control (*β*-actin, HSP90, or GAPDH).

### 4.14. Statistical Analysis

All comparisons between two groups were analyzed by unpaired, two-tailed Student's *t*-tests, and comparisons among three groups were analyzed by one-way ANOVA. Differences were indicated as statistically significant when *P* < 0.05. The data were analyzed using GraphPad Prism (v9.0.2).

## Data Availability

RNA-Seq and ChIP-seq data have been deposited at GEO and are publicly available as of the date of publication. Accession numbers are PRJNA792226, PRJNA790464, and PRJNA798581. All data, materials, and methods will be shared by Prof. Sun and Li upon reasonable request.

## Supplementary Materials

Supplementary Materials Materials and Methods: key resource table. Figure S1: immunoblots of FXR in lung, kidney, heart, spleen, and adipose tissues from FXRf/f and FXRacinar*Δ*/*Δ* mice. Figure S2: validation of FXR deletion in PEOs and establishment of palmitic acid-induced stress models in PEOs. Figure S3: molecular characteristics of PA-induced PEOs injury. Figure S4: RNA-seq on WT and FXR KO PEOs with or without GW4064 treatment. Figure S5: ChIP-seq analysis on WT and FXR KO PEOs with or without GW4064. Figure S6: OSGIN1 interacted with CCT complex and mediated autophagy promoting effects of FXR activation. Table S1: patient information. Table S2: primers for RT-qPCR. Table S3: primers for ChIP -qPCR.
