# Supplementary material for: Activation of Pancreatic Acinar FXR Protects against Pancreatitis via Osgin1-Mediated Restoration of Efficient Autophagy
Source: Research (Wash D C). 2022 Nov 2;2022:9784081. doi: 10.34133/2022/9784081 (PMC9667885; doi:10.34133/2022/9784081)
Supplement: Supplementary Materials — Materials and Methods: key resource table. Figure S1: immunoblots of FXR in lung, kidney, heart, spleen, and adipose tissues from FXRf/f and FXRacinarΔ/Δ mice. Figure S2: validation of FXR deletion in PEOs and establishment of palmitic acid-induced stress models in PEOs. Figure S3: molecular characteristics of PA-induced PEOs injury. Figure S4: RNA-seq on WT and FXR KO PEOs with or without GW4064 treatment. Figure S5: ChIP-seq analysis on WT and FXR KO PEOs with or without GW4064. Figure S6: OSGIN1 interacted with CCT complex and mediated autophagy promoting effects of FXR activation. Table S1: patient information. Table S2: primers for RT-qPCR. Table S3: primers for ChIP -qPCR. [file 9784081.f1.docx]

**Supplemental materials**

**Activation of Pancreatic Acinar FXR Protects Against Pancreatitis via Osgin1-mediated Restoration of Efficient Autophagy**

Yufan Zheng, Wenrui Sun, Zhengyang Wang, Cong Shan, Jiaying Liu, Chenxi He, Borui Li, Xiao Hu, Wenjia Zhu, Liyan Liu, Fei Lan, Changtao Jiang, Chao Zhao, Xiaobo Li, and Ning Sun

**Materials and Methods**

**Key Resource Table**

| REAGENT or RESOURCE | SOURCE | IDENTIFIER |
| --- | --- | --- |
| ANTIBODIES | | |
| Anti-SQSTM1 / p62 Antibody [2C11] - BSA and Azide free | Abcam | Cat# 56416 |
| Rabbit Polyclonal Anti-Myeloperoxidase Antibody | Abcam | Cat# ab9535 |
| Anti-F4/80 Antibody | Abcam | Cat# ab100790 |
| Rabbit Anti-OSGIN1 Antibody (for IHC) | Bioss | Cat# bs-5723R |
| HSP90 Antibody | Cell Signaling Technology | Cat# 4874 |
| LC3A/B (D3U4C) XP® Rabbit Monoclonal Antibody | Cell Signaling Technology | Cat# 12741T |
| TBP Antibody | Cell Signaling Technology | Cat# 8515 |
| Peroxidase-conjugated AffiniPure Donkey Anti-Rabbit IgG | Jackson ImmunoResearch | Cat# 711-035-152 |
| Beta Actin Monoclonal Antibody | ProteinTech | Cat# 66009-1-lg |
| GAPDH Monoclonal Antibody | ProteinTech | Cat# 60004-1-lg |
| HRP-conjugated Affinipure Goat Anti-Mouse IgG(H+L) | ProteinTech | Cat# SA00001-1 |
| HRP-conjugated Affinipure Goat Anti-Rabbit IgG(H+L) | ProteinTech | Cat# SA00001-2 |
| OSGIN1 Polyclonal Antibody | ProteinTech | Cat# 15248-1-AP |
| Anti-NR1H4 antibody produced in rabbit | Sigma Aldrich | Cat# AV33672 |
| FXR Monoclonal Antibody | ThermoFisher | Cat# 417200 |
| Chemicals, peptides, and recombinant proteins | | |
| RIPA lysis buffer | Beyotime | Cat# P0013B |
| Matrigel® Growth Factor Reduced (GFR) Basement Membrane Matrix, Phenol Red-free, LDEV-free | Corning | Cat# 356231 |
| Phosphate Buffered Saline | Corning | Cat# 21-040-CV |
| Penicillin Streptomycin (10000 U/ml) | Gibico | Cat# 15140163 |
| (Z)-Guggulsterone (ZGG) | MedChemExpress | Cat# HY-110066 |
| Chenodeoxycholic Acid (CDCA) | MedChemExpress | Cat# HY-76847 |
| GW4064 | MedChemExpress | Cat# HY-50108 |
| Taurocholic acid (TCA) | MedChemExpress | Cat# HY-B1788 |
| Taurodeoxycholate sodium salt (TDCA) | MedChemExpress | Cat# HY-128853 |
| Cholic acid (CA) | Sigma Aldrich | Cat# C1129 |
| Glycoursodeoxycholic acid (GUDCA) | Sigma Aldrich | Cat# 64480-66-6 |
| Palmitic acid | Sigma Aldrich | Cat# P0500 |
| Sodium taurochenodeoxycholate (TCDCA) | Sigma Aldrich | Cat# T6260 |
| Tamoxifen | Sigma Aldrich | Cat# T5648 |
| Collegenase IV | STEMCELL Technologies | Cat# 07909 |
| Dispase | STEMCELL Technologies | Cat# 07923 |
| DMEM/F-12 with 15 mM HEPES | STEMCELL Technologies | Cat# 36254 |
| DNase I solution (1 mg/ml) | STEMCELL Technologies | Cat# 07900 |
| PancreaCultTM Organoid Growth Medium (Mouse) | STEMCELL Technologies | Cat# 06040 |
| Tauro-β-muricholic acid (T-βMCA) | Steraloids | Cat# 1899-000 |
| 4',6-diamidino-2-phenylindole (DAPI) solution | ThermoFisher | Cat# 62248 |
| Advanced DMEM/F-12 | ThermoFisher | Cat# 12634010 |
| TRIzol reagent | ThermoFisher | Cat# 15596-026 |
| InStab™ Protease Inhibitor Cocktail, EDTA-free, 100×DMSO Stock Solution | Yeasen Biotechnology | Cat# 20124ES03 |
| Phenylmethanesulfonyl fluoride | Yeasen Biotechnology | Cat# 20104ES03 |
| Critical commercial assays | | |
| Nuclear and Cytoplasmic Protein Extraction Kit | Beyotime | Cat# P0027 |
| TrueLib mRNA Library Prep Kit for Illumina | ExCell Bio | Cat# NGS00-2012 |
| Amylase assay kit | Nanjing Jiancheng Bioengineering Institute | Cat# C016-1-1 |
| Lipase assay kit | Nanjing Jiancheng Bioengineering Institute | Cat# A054-2-1 |
| CellTiter-Glo^®^ Luminescent Cell Viability Assay | Promega | Cat# 7572 |
| PCR purification kit | Qiagen | Cat# 28104 |
| Protein A/G agarose beads | Santa | Cat# sc-2003 |
| DAB (SA-HRP) Tunel Cell Apoptosis Detection Kit | Servicebio | Cat# G1507 |
| Diaminobenzidine Chromogenic Kit | Servicebio | Cat# G1212 |
| Diaminobenzidine Chromogenic Kit | Servicebio | Cat# G1212-200T |
| Pierce ECL Western Blotting kit | Tanon | Cat# 180-5001 |
| Neon™ Transfection System kit | ThermoFisher | Cat# MPK1096 |
| ChamQ SYBR Color qPCR Master Mix (Without ROX) | Vazyme | Cat# Q42-02 |
| HiScript III RT SuperMix for qPCR (+gDNA wiper) | Vazyme | Cat# R323-01 |
| VAHTS Universal DNA Library Prep Kit | Vazyme | Cat# ND607 |
| Virus strain: AAV | | |
| AAV-pan-CMV-Osgin1-Gfp | BrainVT A (Wuhan) Co., Ltd. | N/A |
| AAV-pan-CMV-Gfp | BrainVT A (Wuhan) Co., Ltd. | N/A |
| Experimental models: Pancreatic exocrine orgnoids | | |
| Normal pancreatic exocrine orgnoids | The laboratory of Dr. Ning Sun | N/A |
| WT pancreatic exocrine orgnoids | The laboratory of Dr. Ning Sun | N/A |
| FXR KO pancreatic exocrine orgnoids | The laboratory of Dr. Ning Sun | N/A |
| Experimental models: Organisms/strains | | |
| C57BL/6 | Shanghai Jihui Laboratory Animal Care Co.,Ltd. | N/A |
| FXR f/f | The laboratory of Dr. Changtao Jiang | N/A |
| Mist1 creERT2 | The Jackson Laboratory | Strain# 029228 |
| PDX1-iCre | GemPharmatech | Strain# T007670 |
| Software and algorithms | | |
| GraphPad Prism | GraphPad Software | https://www.graphpad.com/scientific-software/prism/ |
| Integrative Genomics Viewer | N/A | http://www.igv.org |
| RStudio | N/A | RRID:SCR_000432 |
| ImageJ v1.50 | National Institute of Health | https://imagej.nih.gov/ij/download.html |
| GSEA v4.2.0 Mac App | UC San Diego and Broad Institute | http://www.gsea-msigdb.org/gsea |

**Figures:**


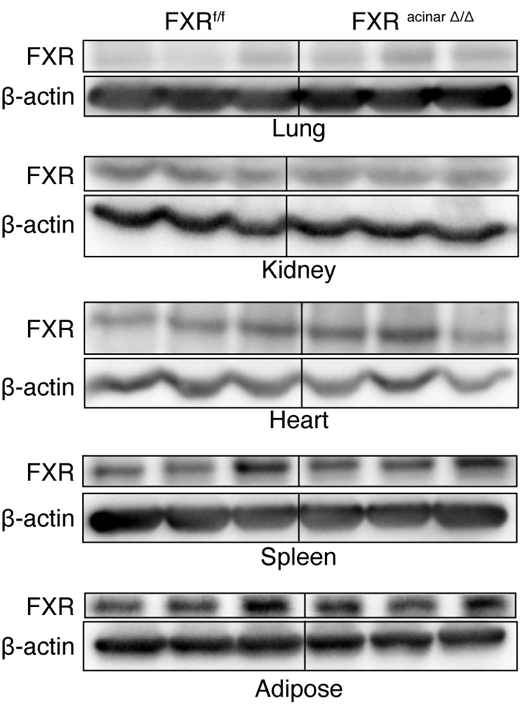


**Figure S1: Immunoblots of FXR in lung, kidney, heart, spleen, and adipose tissues from FXR^f/f^ and FXR^acinarΔ/Δ^ mice.**

**
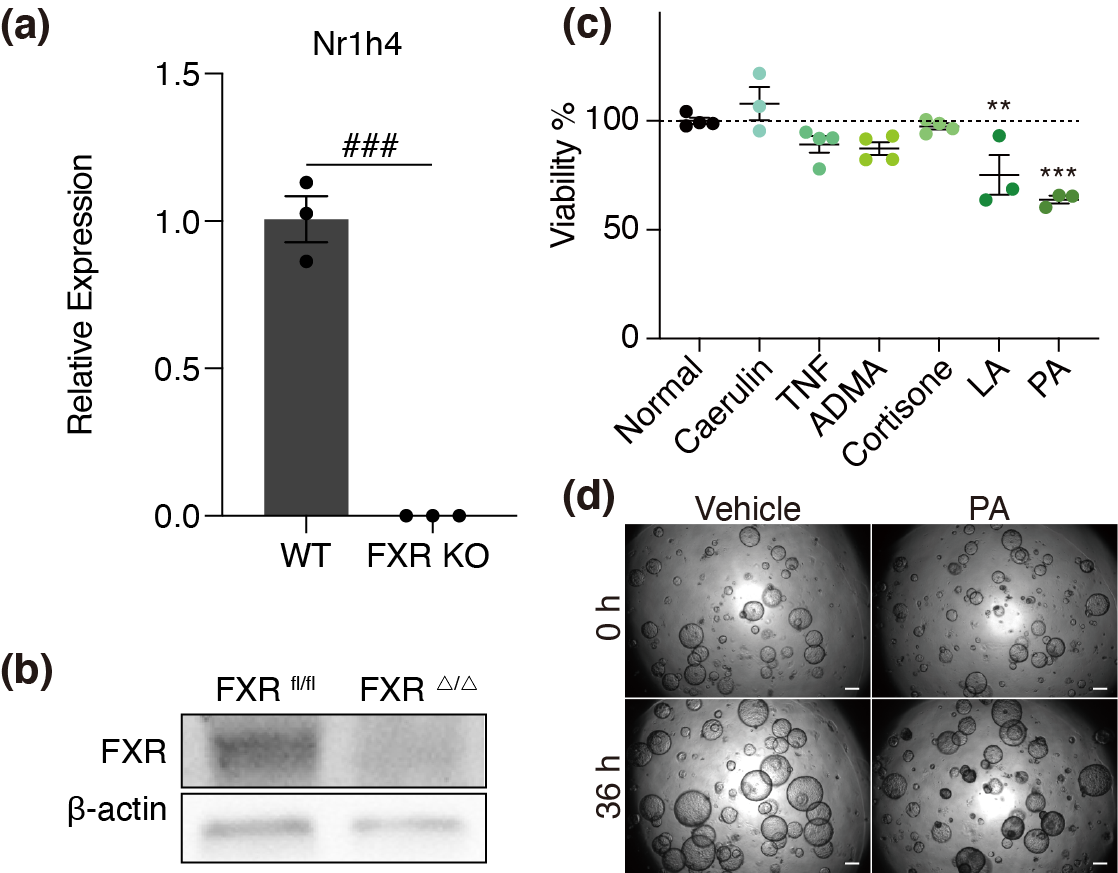
**

**Figure S2: Validation of FXR deletion in PEOs and establishment of palmitic acid-induced stress models in PEOs.** (a and b) qPCR (a) and immunoblot (b) analysis of FXR in WT and FXR KO PEOs. (c) Viability of PEOs treated with 100 nM caerulein, 50 ng/mL tumor necrotic factor α (TNFα), 100 μM asymmetric dimethylarginine (ADMA), 100 nM cortisone, 100 μM linoleic acid (LA), and 100 μM palmitic acid (PA). Viability was normalized with vehicle group respectively. (d) Microscopy images of PEOs at 0 h or 36 h after PA or vehicle treatment. Scale bars, 200 μm. ^###^*P* < 0.001 by Student’s t test. ^**^*P* < 0.01 and ^***^*P* < 0.001 by one-way ANOVA.


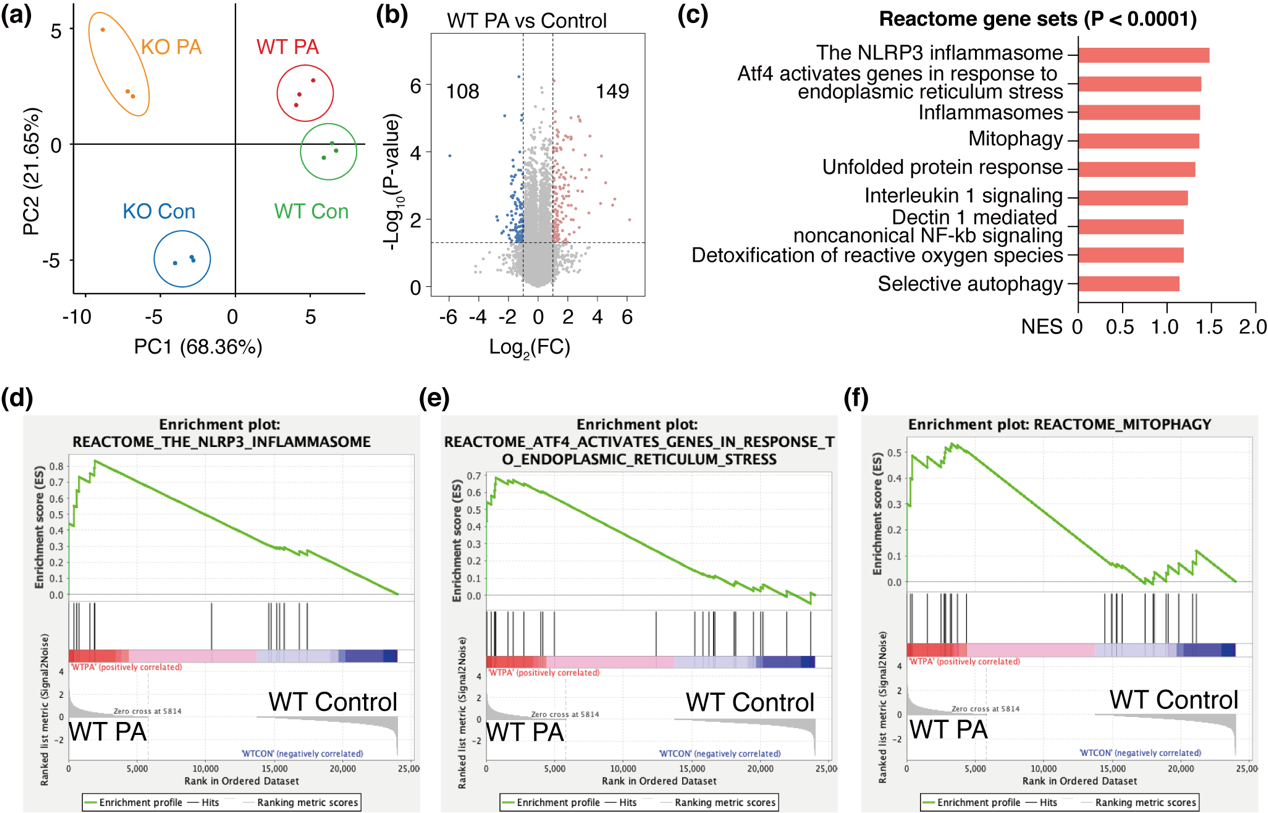


**Figure S3: Molecular characteristics of PA-induced PEOs injury.** (a) Principal component analysis (PCA) of transcriptome of WT and FXR KO PEOs with or without PA treatment. (b) Volcano plot of differentially expressed genes between PA- and vehicle-treated WT PEOs. (c) Gene set enrichment analysis (GSEA) of up-regulated genes in PA-treated WT PEOs compared to vehicle-treated WT PEOs based on Reactome gene set. (d-f) Representatice GSEA plots.


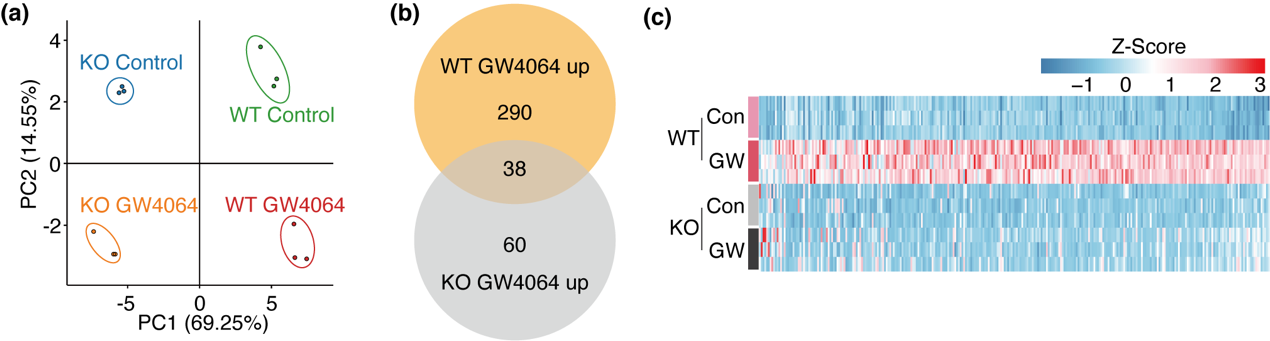


**Figure S4: RNA-seq on WT and FXR KO PEOs with or without GW4064 treatment.** (a) PCA of transcriptome in WT and FXR KO PEOs with or without GW4064 treatment. (b) Venn analysis of up-regulated genes between WT and FXR KO PEOs upon GW4064 treatment. (c) Heatmap of genes up-regulated in WT PEOs but not in FXR KO PEOs after PA treatment.

**
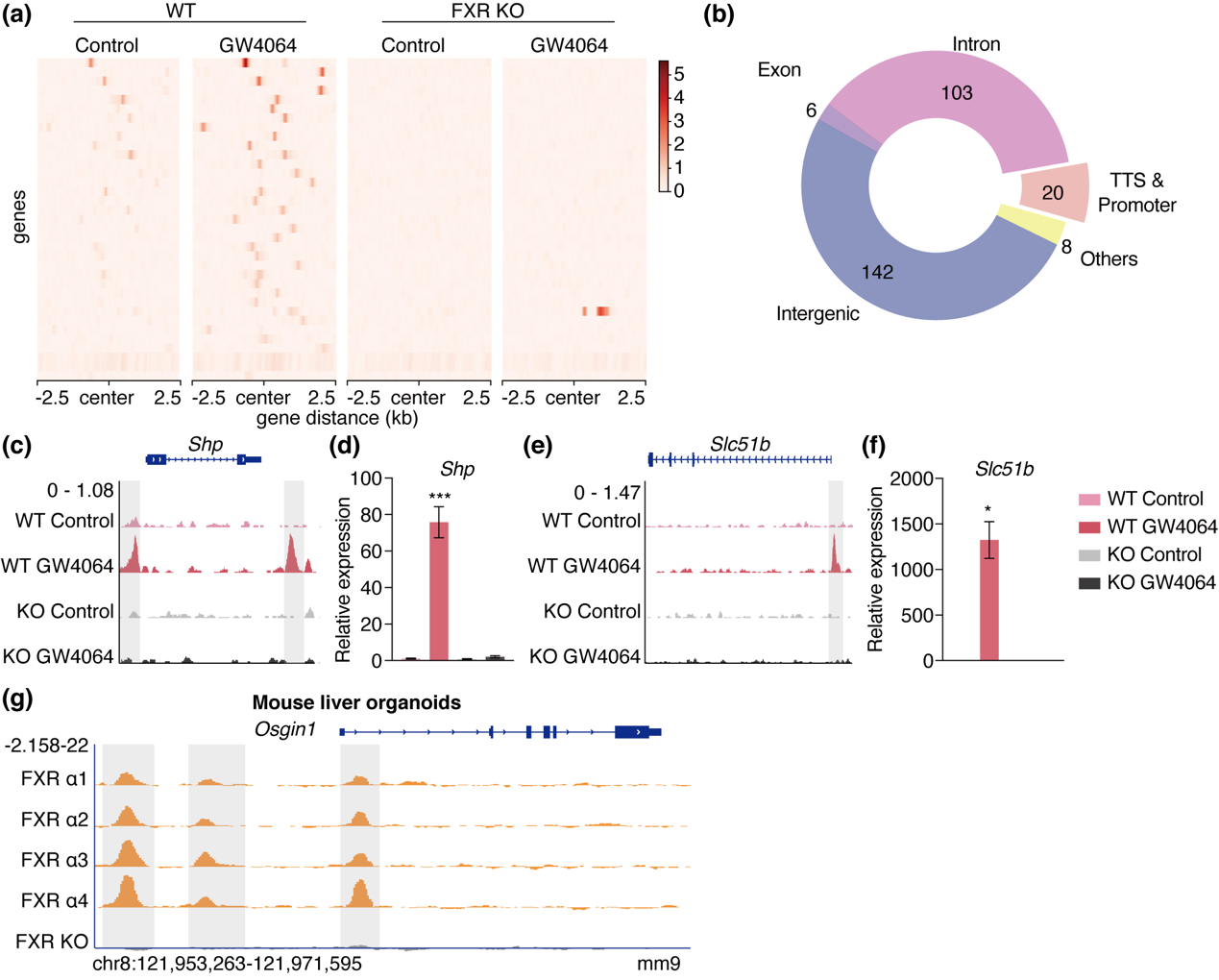
**

**Figure S5: ChIP-seq analysis on WT and FXR KO PEOs with or without GW4064.** (a) Heatmap of FXR occupancy in ChIP-seq peaks in WT and FXR KO PEOs with or without GW4064. (b) FXR binding areas. (c) FXR bound to upstream of Shp to promote its transcriptional expression (d) in WT PEOs but not in FXR KO PEOs. (e) FXR bound to upstream of Slc51b to promote its transcriptional expression (f) in WT PEOs but not in FXR KO PEOs. (g) Four isoforms of FXR bound to the upstream of Osign1 in mouse liver organoids (GSE133700). ^*^*P* < 0.05 and ^***^*P* < 0.001 by one-way ANOVA compared to its own control group.


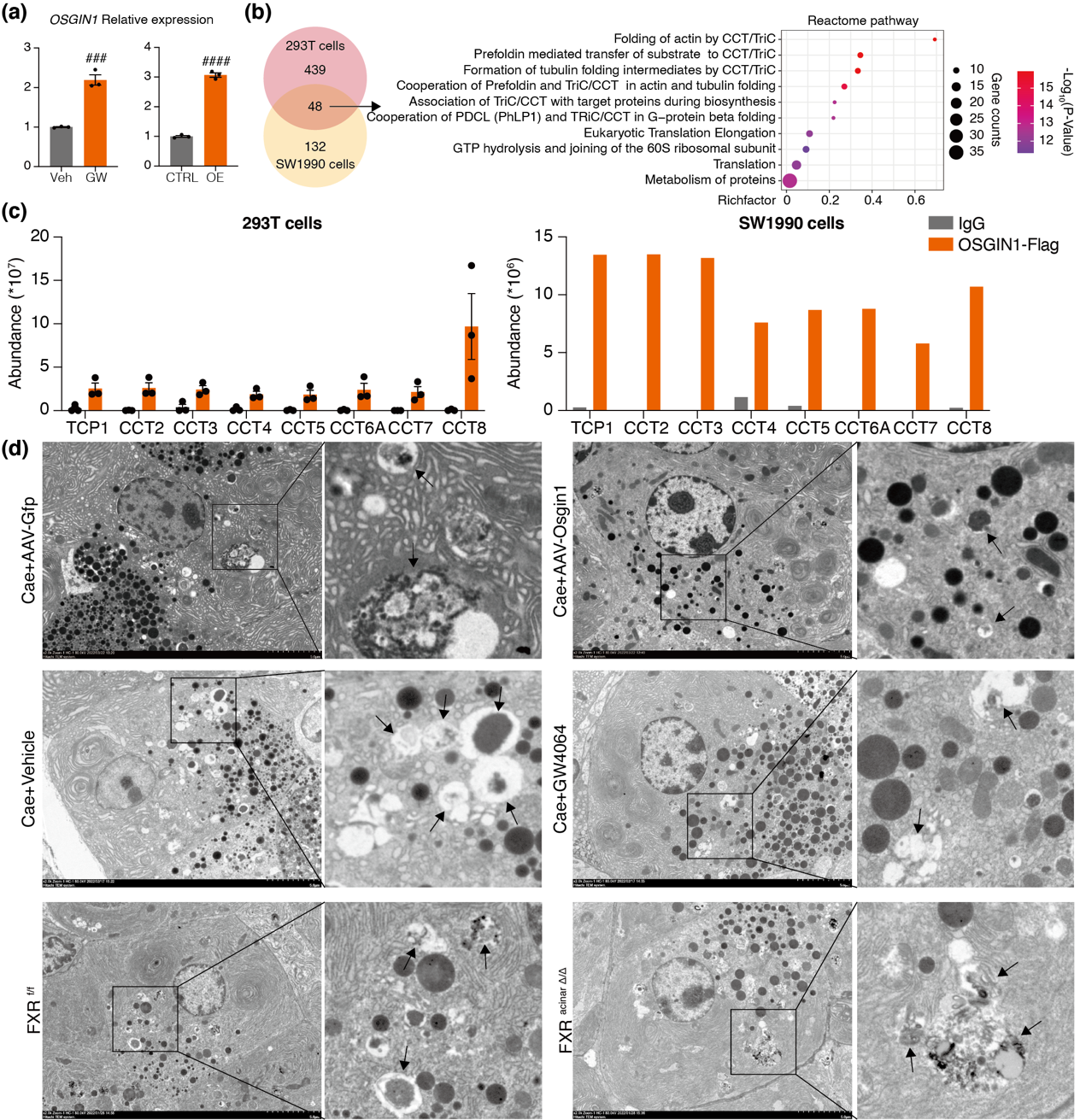


**Figure S6: FXR-OSGIN1 axis regulated autophagy.**

(c) CCT subunits protein abundance Co-IP by osgin1 anti-body in 293T cells and SW1990 cells. (d)Transmission electron microscopy images of mouse pancreatic tissues.

**Table S1: Patient information**

| Patient | Sex/Age | Clinical diagnosis | Tissues type | Serum amylase (U/L) | Serum Lipase (U/L) |
| --- | --- | --- | --- | --- | --- |
|  |  |  |  | Normal range: 0-200 | Normal range: 13-60 |
| 1 | M/45 | Pancreatic adenocarcinoma | Paracancer normal tissues | 67 | 47.6 |
| 2 | M/64 | Pancreatic adenocarcinoma | Paracancer normal tissues | 135 | 245.6 |
| 3 | M/56 | Pancreatic adenocarcinoma | Paracancer normal tissues | NA | NA |
| 4 | M/57 | Pancreatic adenocarcinoma | Paracancer normal tissues | 53 | 10.6 |
| 5 | M/56 | Pancreatic adenocarcinoma | Paracancer normal tissues | NA | NA |
| 6 | M/68 | Pancreatic adenocarcinoma | Paracancer normal tissues | NA | NA |
| 7 | M/60 | Pancreatic ductal adenocarcinoma | Paracancer normal tissues | 15 | 13 |
| 8 | M/48 | Pancreatic adenocarcinoma | Paracancer normal tissues | 80 | 110.5 |
| 9 | M/65 | Pancreatic adenocarcinoma | Paracancer normal tissues | 56 | 59.2 |
| 10 | F/75 | Pancreatic adenocarcinoma | Paracancer normal tissues | 40 | NA |
| 11 | F/35 | Chronic pancreatitis | Pancreatitis tissues | 58 | 75.9 |
| 12 | M/70 | Chronic pancreatitis | Pancreatitis tissues | 58 | 91.2 |
| 13 | M/75 | Chronic pancreatitis | Pancreatitis tissues | 79 | 78.4 |
| 14 | M/51 | Chronic pancreatitis | Pancreatitis tissues | 19 | NA |
| 15 | M/53 | Chronic pancreatitis | Pancreatitis tissues | 14 | 5.5 |
| 16 | M/45 | Chronic pancreatitis | Pancreatitis tissues | 54.75 | 9.5 |
| 17 | M/41 | Autoimmune pancreatitis | Pancreatitis tissues | NA | 7.9 |
| 18 | M/47 | Chronic pancreatitis | Pancreatitis tissues | NA | NA |
| 19 | M/49 | Chronic pancreatitis | Pancreatitis tissues | 18 | 38.9 |
| 20 | M/55 | Autoimmune pancreatitis | Pancreatitis tissues | 31 | 40.7 |
| 21 | M/49 | Chronic pancreatitis | Pancreatitis tissues | 160 | 271.6 |
| 22 | M/57 | Chronic pancreatitis | Pancreatitis tissues | 13 | 4.7 |
| 23 | M/35 | Acute pancreatitis | Pancreatitis tissues | 283 | 98 |
| 24 | M/53 | Chronic pancreatitis | Pancreatitis tissues | 89 | 121.7 |
| 25 | M/53 | Autoimmune pancreatitis | Pancreatitis tissues | 50 | NA |

**Table S2: Primers for RT-qPCR**

| Name | Forward | Reversed |
| --- | --- | --- |
| mouse Fxr (last exon) | CCCTGCTTGATGTGCTAC | GTGATGGTTGAATGTCCG |
| mouse Actb (B actin) | GTGACGTTGACATCCGTAAAGA | GCCGGACTCATCGTACTCC |
| mouse 36b4 | GGGCATCACCACGAAAATCTC | CTGCCGTTGTCAAACACCT |
| mouse Fosb | TTTTCCCGGAGACTACGACTC | GTGATTGCGGTGACCGTTG |
| mouse Cxcl16 | CCTTGTCTCTTGCGTTCTTCC | TCCAAAGTACCCTGCGGTATC |
| mouse Mmp15 | CCGCTGCTACTGGTGCTTC | CATCCACGTTTTCGTCTCTTCAT |
| mouse Ccna2 | TGGATGGCAGTTTTGAATCACC | CCCTAAGGTACGTGTGAATGTC |
| mouse Ccnb1 | AAGGTGCCTGTGTGTGAACC | GTCAGCCCCATCATCTGCG |
| mouse Ccnb2 | GCCAAGAGCCATGTGACTATC | CAGAGCTGGTACTTTGGTGTTC |
| mouse Cdk1 | AGAAGGTACTTACGGTGTGGT | GAGAGATTTCCCGAATTGCAGT |
| mouse Chek2 | TGACAGTGCTTCCTGTTCACA | GAGCTGGACGAACCCTGATA |
| mouse Mcm7 | AGTATGGGACCCAGTTGGTTC | GCATTCTCGCAAATTGAGTCG |
| mouse Shp | TGGGTCCCAAGGAGTATGC | GCTCCAAGACTTCACACAGTG |
| mouse Slc51b | GAGAAACCAGACTTGGCCCC | TCTCAACGCTCCCTAGCTCT |
| mouse Osgin1 | CCTCCGGTATCTGCCTGTC | GGAAAGGTACTCTAGGTCCTGG |
| Human OSGIN1 | CCCACAGGGTAATGGGTGTC | CCCAGACCCTTCTTGACCAC |

**Table S3: Primers for ChIP -qPCR**

| Name | Forward | Reversed |
| --- | --- | --- |
| mouse ChIP Osgin1 [-6592 to -6774] | CGAGGCAACAAACAACAG | TCCTACTTCCATTCCAACG |
| mouse ChIP Osgin1[ -6518 to -6331] | TACCTCGGTCCTCGTTCC | ACATCGGCTAAGTCCTGGG |
| mouse ChIP Osgin1 [-63 to -243] | CCACTCACTGGTAAGGAAGA | GGGACCAGGAAAATCACT |
